# SDF-1/CXCR4 axis enhances the immunomodulation of human endometrial regenerative cells in alleviating experimental colitis

**DOI:** 10.1186/s13287-019-1298-6

**Published:** 2019-07-08

**Authors:** Xiang Li, Xu Lan, Yiming Zhao, Grace Wang, Ganggang Shi, Hongyue Li, Yonghao Hu, Xiaoxi Xu, Baoren Zhang, Kui Ye, Xiangying Gu, Caigan Du, Hao Wang

**Affiliations:** 1 Department of General Surgery, Tianjin Medical University General Hospital, 154 Anshan Road, Heping District, Tianjin, 300052 China; 2 Tianjin General Surgery Institute, Tianjin, China; 3 Xiyuan Hospital, China Academy of Chinese Medical Sciences, Beijing, China; 4 Faculty of Medicine, University of Toronto, Toronto, Ontario Canada; 5 Department of Colorectal Surgery, The Second Hospital of Tianjin Medical University, Tianjin, China; 6 Department of Endocrinology, Tianjin Medical University General Hospital, Tianjin, China; 7 Department of Vascular Surgery, Tianjin Fourth Central Hospital, Tianjin, China; 8 Department of Urologic Sciences, The University of British Columbia, Vancouver, British Columbia Canada; 9 Immunity and Infection Research Centre, Vancouver Coastal Health Research Institute, Vancouver, British Columbia Canada

**Keywords:** Endometrial regenerative cells, Stromal cell-derived factor-1, C-X-C chemokine receptor type 4, Immunoregulation

## Abstract

Endometrial regenerative cells (ERCs) are a new type of mesenchymal-like stromal cells, and their therapeutic potential has been tested in a variety of disease models. SDF-1/CXCR4 axis plays a chemotaxis role in stem/stromal cell migration. The aim of the present study was to investigate the role of SDF-1/CXCR4 axis in the immunomodulation of ERCs on the experimental colitis. The immunomodulation of ERCs in the presence or absence of pretreatment of SDF-1 or AMD3100 was examined in both in vitro cell culture system and dextran sulphate sodium-induced colitis in mice. The results showed that SDF-1 increased the expression of CXCR4 on the surface of ERCs. As compared with normal ERCs, the SDF-1-treated, CXCR4 high-expressing ERCs more significantly suppressed dendritic cell population as well as stimulated both type 2 macrophages and regulatory T cells in vitro and in vivo. Meanwhile, SDF-1-pretreated ERCs increased the generation of anti-inflammatory factors (e.g., IL-4, IL-10) and decreased the pro-inflammatory factors (e.g., IL-6, TNF-α). In addition, SDF-1-pretreated CM-Dil-labeled ERCs were found to engraft to injured colon. Our results may suggest that an SDF-1-induced high level of CXCR4 expression enhances the immunomodulation of ERCs in alleviating experimental colitis in mice.

## Background

Inflammatory bowel disease (IBD) is a chronic disabling inflammatory process that includes ulcerative colitis (UC) and Crohn’s disease (CD), and its damage mainly involves in the ileum, colon, and rectum [1]. Currently, there are high incidence and prevalence of UC in the developed countries, and during the past decades, the numbers of UC are rapidly rising in other parts of the world including China [2]. As of today, the etiopathogenesis of the UC remains largely unknown, but currently, UC is considered as a polygenic autoimmune disorder that is caused by many different genetic factors, environmental factors, intestinal flora (microbiome), or immune response [3–8]. As a type of autoimmune diseases, UC is associated with a dysregulated Th2 response [9]. The current treatment of UC includes 5-amino salicylic acid, glucocorticoids, antibiotics, immunosuppressants, anti-TNF agents, and surgical therapy [10]. However, the outcome of these treatments is not always satisfied. In fact, a certain number of patients are still suffering from either the UC itself or the side effects caused by various treatments [1]. In recent years, mesenchymal stromal cells (MSCs) are considered as a promising cell therapy for the treatment of UC [11]. However, some deficiencies of MSCs limit their usage such as the invasive harvesting procedure and related complications, limited proliferation capacity, and less availability [12]. Therefore, it is necessary to search a new source of regenerative cells, which could both come into a therapeutic effect and overcome the shortcomings of MSCs.

Endometrial regenerative cells (ERCs) are mesenchymal-like stromal cells, which can be isolated from human menstrual blood [12]. ERCs have many advantages over other sources of MSCs including body waste reusage, abundant resources, non-invasively obtained method, pluripotent differentiation activity, anti-inflammatory ability, lack of immunogenicity, expandability to great quantities without karyotypic abnormalities or the loss of differentiation ability, and without tumorigenesis [13–15]. Moreover, a previous study has confirmed that these human ERCs are not rejected in the xenogeneic animal models [16]. We and others have demonstrated that ERCs are excellent candidates for the treatment of numerous experimental disease models, such as prevention or attention of renal ischemia-reperfusion injury [17], acute liver injury [18], and critical limb ischemia [16] in mouse models. Recently, our group has demonstrated that ERCs could attenuate experimental colitis in mice by regulating T and B cell responses [19, 20], as well as by reducing the infiltration of inflammatory cells to the damaged tissues [19]. However, the in-depth mechanisms of ERCs in the treatment of colitis are not well understood, which may be required for further development of ERCs as a novel cell therapy to alleviate colitis in patients.

Stromal cell-derived factor-1 (SDF-1) is a key member of the superfamily of chemotactic cytokines that interacts with its receptor chemokine receptor 4 (CXCR4) on the surfaces of the stem/progenitor cells [21]. The SDF-1/CXCR4 axis not only plays important roles in stem/stromal cells mobilization, proliferation, migration, adhesion, survival, and paracrine, but also it is required for the therapeutic effect of stem/stromal cell-based therapies [22–24]. Moreover, evidence in the literature has demonstrated that ERCs could secrete SDF-1 and express CXCR4 [21, 25]. Hence, the aim of this study was to determine whether the therapeutic effect of ERCs on the experimental colitis could be determined by the SDF-1/CXCR4 axis via pretreatment of ERCs with SDF-1.

## Methods

### Isolation and culture of ERCs

Human ERCs were isolated from the menstrual blood of healthy women who were 20–30 years old. The procedure was ethically approved by Tianjin Medical University General Hospital (Tianjin, China). A volume of 5 ml of menstrual blood from each consented donor was collected by using a sterilized menstrual cup in an antibiotic-containing solution. As described previously [26], the mononuclear cells from the menstrual blood were obtained by using a standard Ficoll method, followed by suspension in high glucose Dulbecco’s modified Eagle’s medium (DMEM) containing 10% fetal bovine serum (FBS) and 1% penicillin/streptomycin. The cell suspension was divided into two parts/10 cm dishes that were cultured in a 37 °C 5% CO_2_ incubator. After overnight incubation, the non-adherent cells were removed, and the remaining adherent cells were incubated for 2 weeks when the cells displayed a spindle-shaped morphology.

### Determination of CXCR4 expression on ERCs

To confirm the effect of SDF-1 on stimulating the expression of CXCR4 (CD184) on ERCs, the ERCs were seeded onto a 24-well plate (5 × 10^4^ cells/well) and cultured with different concentrations of SDF-1 (0, 10, 30, 50, 100 ng/ml) for 72 h at 37 °C (*n* = 6). Then, the ERCs were collected and labeled with anti-CXCR4 antibodies (anti-CD184-PE, BioLegend, San Diego, USA). The percentages of CXCR4^+^ ERCs were measured by using a flow cytometric analysis.

### Co-cultures of ERCs and allogeneic splenocytes

To investigate whether SDF-1-pretreated ERCs could affect the differentiation of allogeneic splenocytes, ERCs and splenocytes of BALB/c were co-cultured in a 96-well plate and stimulated with various stimulators for 96 h. In brief, ERCs (1 × 10^4^ cells) pretreated with or without SDF-1 (50 ng/ml)/AMD3100 (CXCR4 antagonist, 1 μg/ml, Sigma-Aldrich, St. Louis, USA) were co-cultured with splenocytes (2 × 10^5^ cells). The lipopolysaccharide (LPS, 10 μg/ml, Solarbio, Beijing, China) was used to stimulate the generation of dendritic cells (DCs). The anti-mouse CD3 (100 ng/ml) and CD28 (200 ng/ml) antibodies (*e*Bioscience, San Diego, USA) were used to stimulate the generation of regulatory T cells (Tregs). For type 2 macrophages (M2), stimulators were interleukin (IL)-4 (100 U/ml) and LPS (10 μg/ml) (Peprotech, Rocky Hill, USA). The percentages of each cell types were examined by using flow cytometric analysis (*n* = 6/each group).

### Animals

Male adult BALB/c mice (18–20 g bodyweight, 6–8 weeks old) were purchased from the China Food and Drug Inspection Institute (Beijing, China). The mice were housed under a conventional experimental environment in the animal facility at Tianjin General Surgery Institute (Tianjin, China) and provided with water and chow ad libitum. All the experiments were performed according to the Chinese Council on Animal Care guidelines and the protocols approved by the Animal Care and Use Committee of Tianjin Medical University (Tianjin, China).

### Experimental groups

Mice were randomly assigned to five experimental groups (*n* = 6 per group): group 1, normal control; group 2, untreated; group 3, treated with unaltered ERCs (ERCs); group 4, treated with SDF-1-pretreated ERCs (^#^ERCs); and group 5, treated with AMD3100-pretreated ERCs (*ERCs). The mice in the normal control group (group 1) were fed with water for 10 days. In order to induce colitis, the mice, except the normal control group, were fed with water containing 3% dextran sulphate sodium (DSS) *w*/*v* (3% *w*/*v*) (MP Biomedicals, Santa Ana, USA) for 7 days (days 1–7) and then administered water for 3 days (days 8–10) as described previously [27]. The DSS-induced colitis in mice of the untreated group (group 2) was injected with 200 μl PBS at days 2, 5, and 8. Unaltered ERCs (1 × 10^6^ cells/mouse) were suspended in 200 μl PBS and then injected intravenously at days 2, 5, and 8 in the ERC-treated colitis group (group 3). For the SDF-1-pretreated ERCs group (group 4), the ERCs were co-cultured with SDF-1 (50 ng/ml) for 72 h then intravenously injected (1 × 10^6^ cells/mouse) into the BALB/c mice with colitis at days 2, 5, and 8. For the AMD3100-pretreated ERCs group (group 5), the ERCs were co-cultured with the SDF-1 receptor antagonist AMD3100 (5 mg/kg/test) for 30 min then intravenously injected (1 × 10^6^ cells/mouse) into the BALB/c mice with colitis at the same time points as for groups 2–4.

### Assessment of inflammation severity

The mouse clinical signs were recorded, and body weight was monitored daily. The Disease Activity Index (DAI) represented the sum of the scores according to the following standards [28]: (a) body weight loss—0 (no change), 1 (1–5%), 2 (5–10%), 3 (10–20%), and 4 (> 20%); (b) stool consistency—0 (normal), 1 (loose stools), 2 (loose stools), 3 (mild diarrhea), and 4 (watery diarrhea); and (c) hemoccult positivity and the presence of gross stool blood—0 (normal), 1 (positive fecal occult blood), 2 (positive fecal occult blood), 3 (visible rectal bleeding), and 4 (severe rectal bleeding). All of the mice were euthanized on day 10 for sample collection.

### Histology

The colon from the ileocecal junction to the anus was collected, followed by the measurement of its length. The colon samples were fixed in 10% formalin and embedded in paraffin. Then, the hematoxylin and eosin (H&E) staining was performed on the colon sections. The intensity of inflammation, as well as changes in the mucus structure and intestinal epithelium, was examined as described previously [29].

### Flow cytometry analysis

The population of each phenotype of immune cells in different groups was evaluated by using flow cytometric analysis as described previously [30]. In brief, splenocytes were stained with fluorescent antibodies, including anti-CD4-FITC, anti-CD25-PE, anti-Foxp3-PerCP, anti-CD11c-APC, anti-MHCII-FITC, anti-CD86-PE, anti-CD40-PE, anti-CD4-PE (*e*Biosciences, San Diego, USA), anti-IL-4-APC, anti-CD68-FITC, and anti-CD206-PE (BioLegend, San Diego, USA), according to the manufacturer’s instruction. The FlowJo software was used to analyze the percentages of various phenotypes of immune cells.

### Enzyme-linked immunosorbent assay

According to the manufacturer’s instructions, the colon homogenate supernatants of TNF-α, IL-4, IL-6, and IL-10 in BALB/c mice in each group were measured by the ELISA kit (DAKEWE, Shenzhen, China). The optical density (OD) value was measured by using a microplate reader.

### Labeling of SDF-1-pretreated ERCs with CM-Dil and in vivo tracking

For in vivo tracking of administered ERCs pretreated with SDF-1, cells were isolated and labeled with CM-Dil (Thermo Fisher Scientific, Waltham, USA), according to the manufacturer’s instructions. CM-Dil-labeled ERCs were pretreated with SDF-1, followed by injection (1 × 10^6^ cells/mouse) via the tail vein at day 7 after colitis induction. The spleen, colon, and kidney were collected from the colitis mice 24 h later, and 4 μm frozen sections were cut. The CM-Dil-labeled ERCs were localized using the fluorescence microscope.

### Statistical analysis

All the experimental data were expressed as mean ± standard deviation (SD). The differences between multiple groups were analyzed using one-way analysis of variance (ANOVA). Differences with *p* values when *p* < 0.05 were considered significant.

## Results

### SDF-1 increased the expression of CXCR4 on ERCs in vitro

**Fig. 1:**
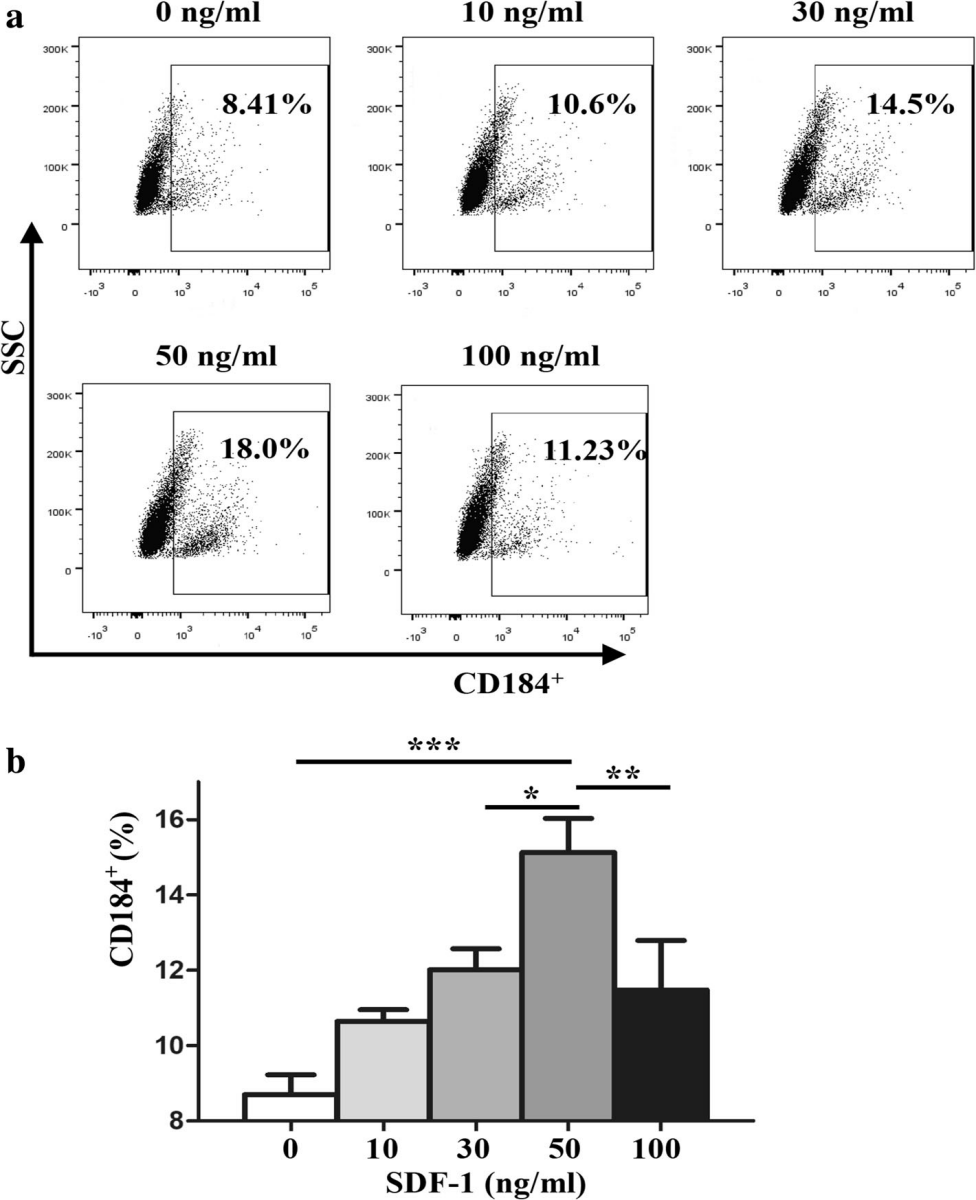
SDF-1 promoted the expression of CXCR4^+^ on the surface of ERCs. **a**, **b** The expression of CXCR4^+^ was analyzed by flow cytometry. The *p* value was determined by one-way ANOVA, *n* = 6. ***p < 0.001, **p < 0.01, *p < 0.05

To validate whether SDF-1 could affect the surface expression of CXCR4 on ERCs, we analyzed and compared the percentages of CXCR4^+^ ERCs after treatment with different concentrations of SDF-1. As shown in Fig. 1, when the concentration of SDF-1 was less than 50 ng/ml, the expression of CXCR4 was increased in an SDF-1 dose-dependent manner (0 ng/ml vs. 50 ng/ml, *p* < 0.001; 30 ng/ml vs. 50 ng/ml, *p* < 0.05). However, the percentage of CXCR4^+^ ERCs decreased obviously when the concentration of SDF-1 increased to 100 ng/ml (*p* < 0.01). Therefore, we chose 50 ng/ml of SDF-1 as an optimal concentration in the following experiments.

### CXCR4 high-expressing ERCs suppressed the generation of DCs and promoted of M2 and Tregs in vitro

**Fig. 2:**
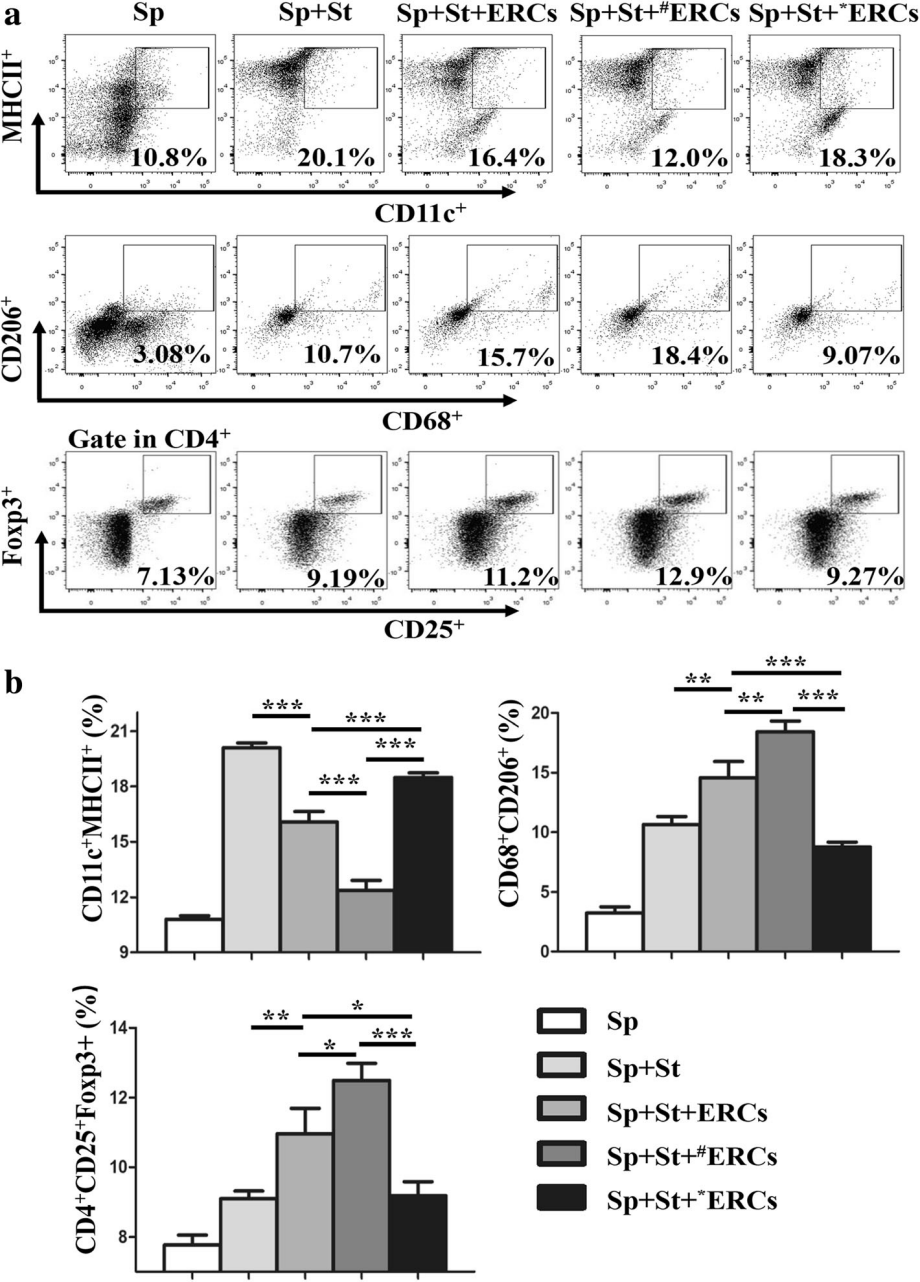
SDF-1 plays a crucial role in ERC-mediated generation of DC, M2, and Treg cells in vitro. **a**, **b** Splenocytes obtained from BALB/c mice were co-cultured with ERCs which were pretreated with or without SDF-1/AMD3100 and different stimulators for 96 h. The percentages of DCs (CD11c^+^MHCII^+^), M2 (CD68^+^CD206^+^), and Tregs (CD4^+^CD25^+^Foxp3^+^) were measured by flow cytometry. Sp indicated splenocytes. St indicated the corresponding stimulators. ^**#**^ERCs indicated ERCs pretreated with SDF-1. *ERCs indicated inhibition the function of SDF-1 by AMD3100. The *p* value was determined by one-way ANOVA, *n* = 6. ***p < 0.001, **p < 0.01, *p < 0.05

The effects of CXCR4 expression of ERCs on the generations of DCs (CD11c^+^ MHCII^+^), M2 (CD68^+^ CD206^+^), and Tregs (CD4^+^ CD25^+^ Foxp3^+^) in an in vitro co-culture experiment were investigated. The percentages of each type of cells were measured by using flow cytometric analysis. As shown in Fig. 2a, co-culturing splenocytes with unaltered ERCs significantly decreased the percentages of DCs (CD11c^+^MHCII^+^) and increased the percentages of M2 and Tregs as compared to co-culturing splenocytes with the stimulators only (DCs, *p* < 0.001; M2, *p* < 0.01; Tregs, *p* < 0.01; Fig. 2b). Further, co-culturing splenocytes with SDF-1-pretreated ERCs further decreased the percentages of DCs compared to co-culture splenocytes with ERCs (DCs, *p* < 0.001; Fig. 2b). Co-culturing splenocytes with SDF-1-pretreated ERCs further increased the percentages of M2 and Tregs, compared to co-culture splenocytes with ERCs (M2, *p* < 0.01; Tregs, *p* < 0.05; Fig. 2b). However, the regulatory effects of ERCs were eliminated when the ERCs were pretreated with AMD3100, as compared with the groups cultured with either unaltered ERCs (DCs, *p* < 0.001; M2, *p* < 0.001; Tregs, *p* < 0.05; Fig. 2b) or SDF-1-pretreated ERCs (DCs, *p* < 0.001; M2, *p* < 0.001; Tregs, *p* < 0.001; Fig. 2b). These data suggest that a high level of CXCR4 expression on ERCs promoted the immunomodulatory effect of ERCs.

### CXCR4 high-expressing ERCs alleviated DSS-induced experimental colitis

**Fig. 3:**
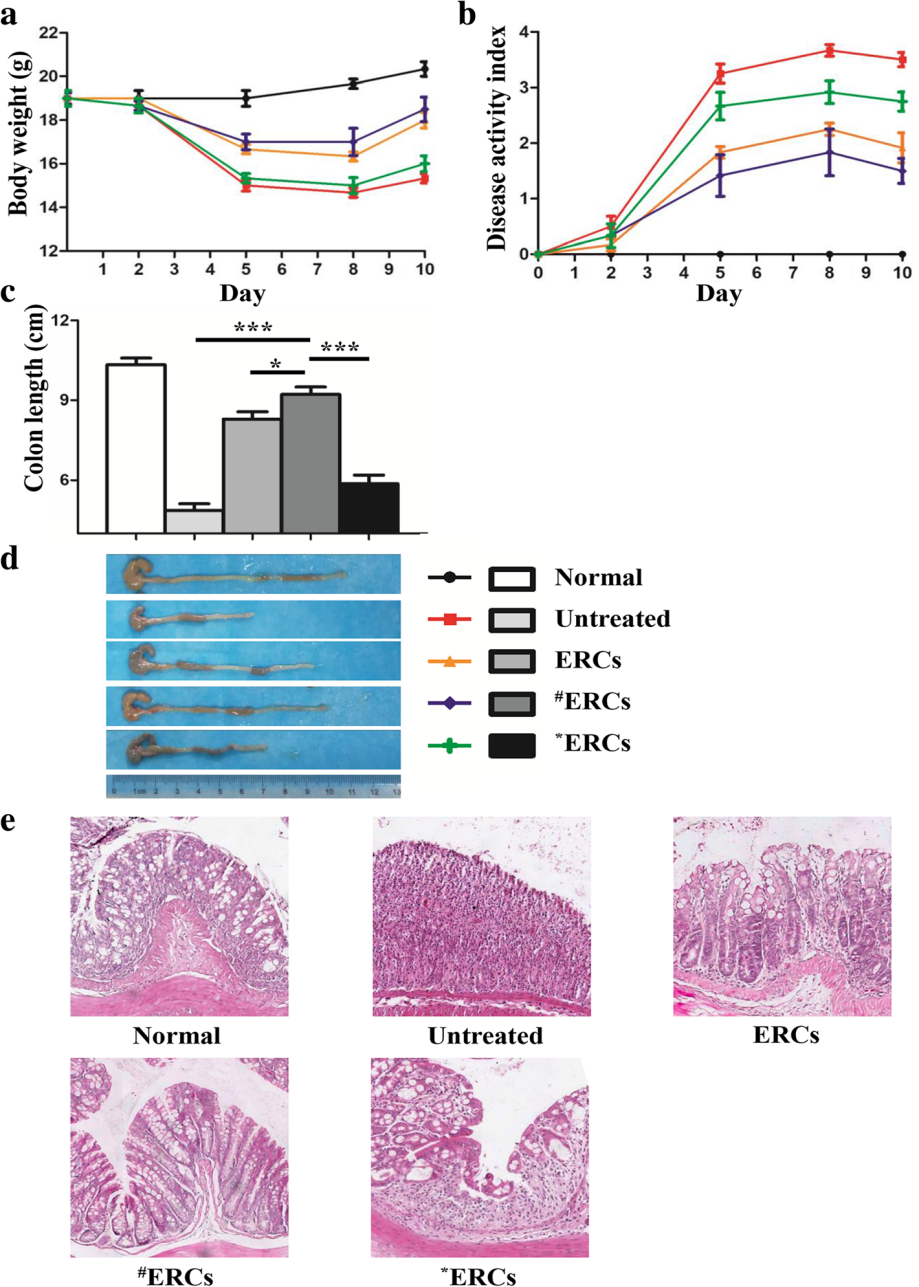
CXCR4 high-expressing ERCs protect against DSS-induced severe colitis. SDF-1-pretreated ERCs (**a**) attenuated the body weight loss and (**b**) alleviated the Disease Activity Index (DAI) of DSS-induced colitis in mice. **c**, **d** Mice were sacrificed at day 10 after DSS induction. The photograph shows colonic specimens from each group of mice. **e** Colon specimens were sectioned and stained with H&E. ^**#**^ERCs indicated ERCs pretreated with SDF-1. *ERCs indicated inhibition of the function of SDF-1 by AMD3100. The *p* value was determined by one-way ANOVA. ***p < 0.001, **p < 0.01, *p < 0.05

In the untreated group, we found that the body weight of the mice decreased significantly during the first 5 days, as described in our previous studies [20]. Compared with the untreated group, the mouse body weights in the unaltered ERC-treated group and SDF-1-pretreated ERCs group were increased to a certain extent. While the mouse body weight in the SDF-1-pretreated ERCs group and unaltered ERCs group recovered significantly between days 8 and 10, as compared to that of the untreated group (Fig. 3a, SDF-1-pretreated ERCs group vs. untreated group: D8, *p* < 0.001; D10, *p* < 0.001; unaltered ERCs group vs. untreated group: D8, *p* < 0.01; D10, *p* < 0.001). On the other hand, treatment with SDF-1-pretreated ERCs remarkably attenuated the severity of experimental colitis and bloody stool (Fig. 3b, compared to the untreated group, D8: *p* < 0.001; D10: *p* < 0.001), as well as protected the length of the colon (Fig. 3c, d, compared to the untreated group, *p* < 0.001). AMD3100 completely blocked the therapeutic effect of ERCs.

In tissue sections with HE staining, as shown in Fig. 3e, the tissue in the control group had a normal structure of the colon. However, the tissue in the untreated colitis group showed massive inflammatory cell infiltration in the mucosa and submucosa, as well as the structures of the epithelium and crypts were damaged. By comparison, the mucosal hyperemia and edema were obviously remitted, and inflammatory cell infiltration was significantly decreased in both the SDF-1-pretreated ERCs group and the unaltered ERC-treated group. In addition, AMD3100 abolished the therapeutic effects of either unaltered ERCs or SDF-1-pretreated ERCs.

### CXCR4 high-expressing ERCs decreased the percentage of DCs in splenocytes

**Fig. 4:**
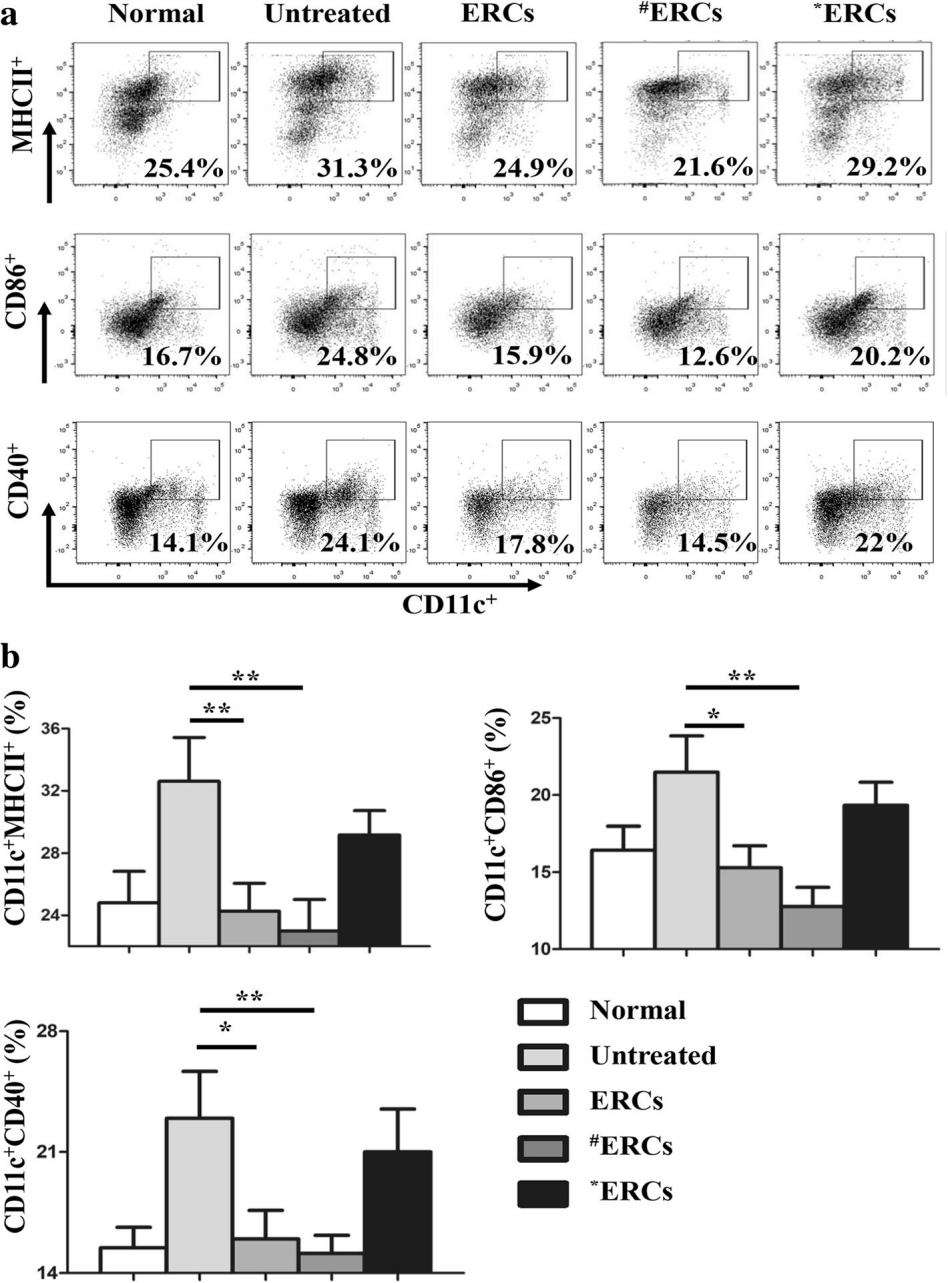
CXCR4 high-expressing ERCs in decreasing the percentage of DC in DSS-induced colitis in mice. The spleen was dissected and made into a single-cell suspension. Cells were stained with fluorescently labeled CD11c, MHCII, CD86, and CD40 and detected by flow cytometry. **a** Dot plots of CD11c^+^MHCII^+^, CD11c^+^CD86^+^, and CD11c^+^CD40^+^ cells. **b** Percentage of CD11c^+^MHCII^+^, CD11c^+^CD86^+^, and CD11c^+^CD40^+^ cells. ^**#**^ERCs indicated ERCs pretreated with SDF-1. *ERCs indicated inhibition of the function of SDF-1 by AMD3100. The *p* value was determined by one-way ANOVA. ***p < 0.001, **p < 0.01, *p < 0.05

To confirm the effects of CXCR4 high-expressing ERCs in vivo, we have analyzed the expression of splenic immune cell populations by flow cytometry on day 10 after colitis induction. The DC population in splenocytes gated by CD11c was investigated through expressing positive MHC II, CD86, and CD40. The results showed that the ERC-treated group had lower population of CD11c^+^MHCII^+^, CD11c^+^CD86^+^, and CD11c^+^CD40^+^ DCs as compared to the untreated group (Fig. 4, CD11c^+^MHCII^+^, *p* < 0.01; CD11c^+^CD86^+^, *p* < 0.05; and CD11c^+^CD40^+^, *p* < 0.05); the SDF-1-pretreated ERCs group had further significantly lower population of CD11c^+^MHCII^+^, CD11c^+^CD86^+^, and CD11c^+^CD40^+^ DCs as compared to the untreated group (Fig. 4, *p* < 0.01). In contrast, AMD3100 blocked the therapeutic function of ERCs, and much more cells of CD11c^+^MHCII^+^, CD11c^+^CD86^+^, and CD11c^+^CD40^+^ DCs were found in colitis mice treated with AMD3100-pretreated ERCs, which were indistinguishable from that of the untreated group (Fig. 4). These data suggest that either unaltered ERCs or SDF-1-pretreated ERCs could significantly inhibit the development of DC.

### CXCR4 high-expressing ERCs increased the percentage of Th2 and Tregs in splenocytes

**Fig. 5:**
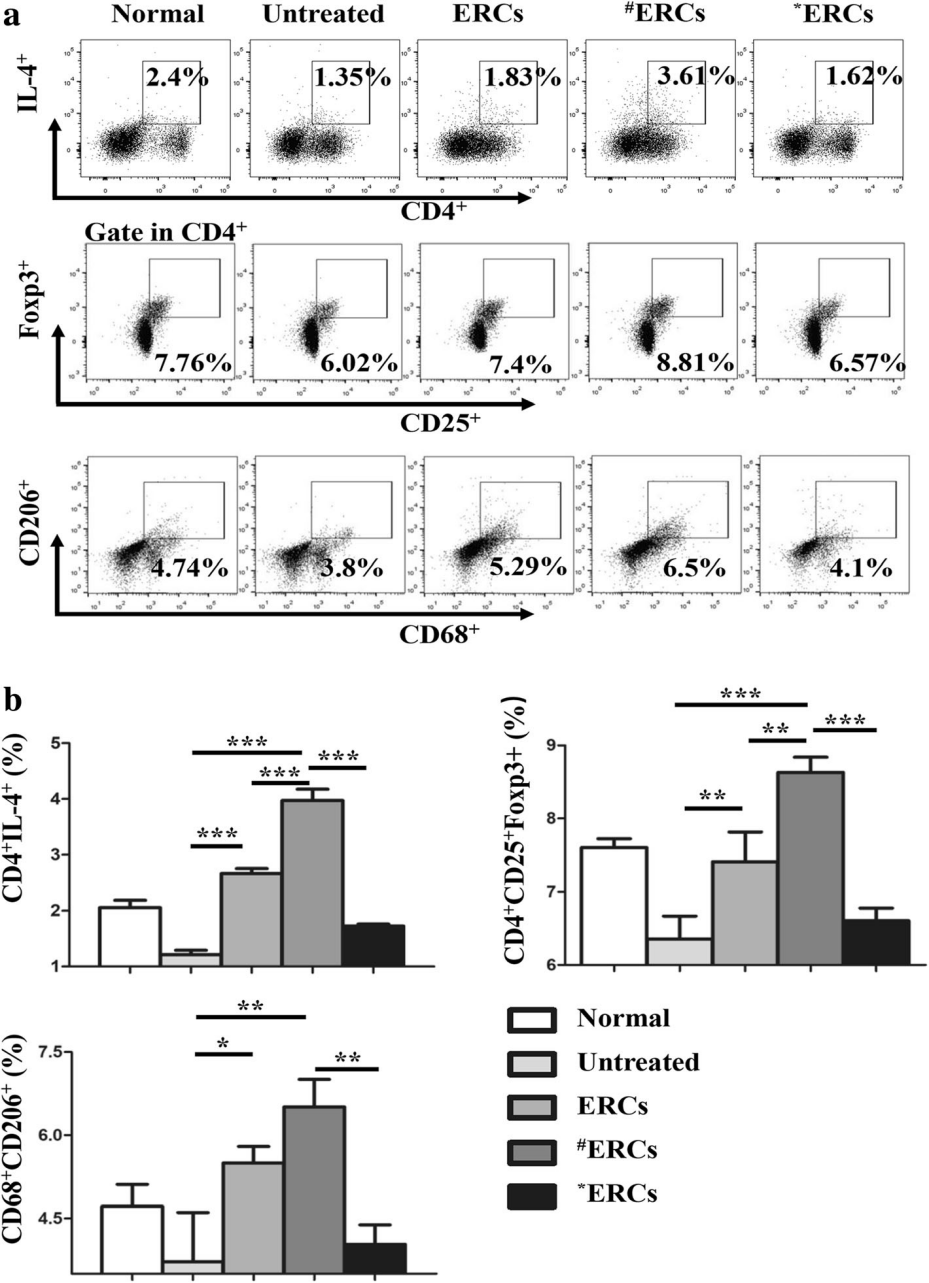
CXCR4 high-expressing ERCs in increasing the percentage of Th2, Tregs, and M2 in DSS-induced colitis in mice. The spleen was dissected and made into a single-cell suspension. Cells were stained with fluorescently labeled CD4, IL-4, CD4, CD25, Foxp3, CD68, and CD206 and detected by flow cytometry. **a** Dot plots of Th2, Tregs, and M2 cells. **b** Percentage of Th2, Tregs, and M2 cells. ^#^ERCs indicated ERCs pretreated with SDF-1. *ERCs indicated inhibition of the function of SDF-1 by AMD3100. The *p* value was determined by one-way ANOVA. ***p < 0.001, **p < 0.01, *p < 0.05

To identify the effect of the SDF-1-pretreated ERCs in DSS-induced colitis in mice, we investigated the percentage of Th2 through double-positive staining of the anti-mouse CD4 and IL-4 antibodies and Tregs through triple-positive staining of the anti-mouse CD4, CD25, and Foxp3 antibodies in the splenocytes of each group by flow cytometry. As shown in Fig. 5, as compared with the untreated group, both the percentages of Th2 and Tregs were increased in the ERC-treated group (Fig. 5, Th2, *p* < 0.001; Treg, *p* < 0.01), and the percentages of Th2 and Tregs were increased in the SDF-1-pretreated ERCs group as compared to either the ERC-treated group or untreated group (Fig. 5, SDF-1-pretreated ERCs group vs. unaltered ERC-treated group: Th2, *p* < 0.001; Treg, *p* < 0.01; SDF-1-pretreated ERCs group vs. untreated group: Th2, *p* < 0.001; Treg, *p* < 0.001). The percentages of Th2 and Tregs were remarkably much higher in the SDF-1-pretreated ERCs group than in the AMD3100-pretreated ERCs group (Fig. 5, Th2, *p* < 0.001; Treg, *p* < 0.001). The effect of ERCs on enhancing Th2 and Treg population was eliminated by the addition of AMD3100 (Fig. 5, AMD3100-pretreated ERCs group vs. unaltered ERC-treated group: Th2, *p* < 0.001; Treg, *p* < 0.05). These data indicate that SDF-1-pretreated ERCs could significantly promote the development of Th2 and Treg.

### CXCR4 high-expressing ERCs increased the percentage of M2 in splenocytes

To examine the effect of different treatments affecting the population of M2, CD68 and CD206 were used to measure M2 phenotype in splenocytes. As shown in Fig. 5, the percentage of M2 in the ERC-treated group was increased compared to the untreated group (*p* < 0.05). The M2 population was further increased in the SDF-1-pretreated ERCs group than the untreated group (Fig. 5, *p* < 0.01). The percentage of M2 was considerably increased in the SDF-1-pretreated ERCs group compared to that in the AMD3100-pretreated ERCs group (Fig. 5, *p* < 0.01), which is indistinguishable from the untreated group. These data imply that SDF-1-pretreated ERCs could significantly promote the development of M2.

### CXCR4 high-expressing ERCs reduced the levels of TNF-α and IL-6 and increased the levels of IL-4 and IL-10 in the colon

**Fig. 6:**
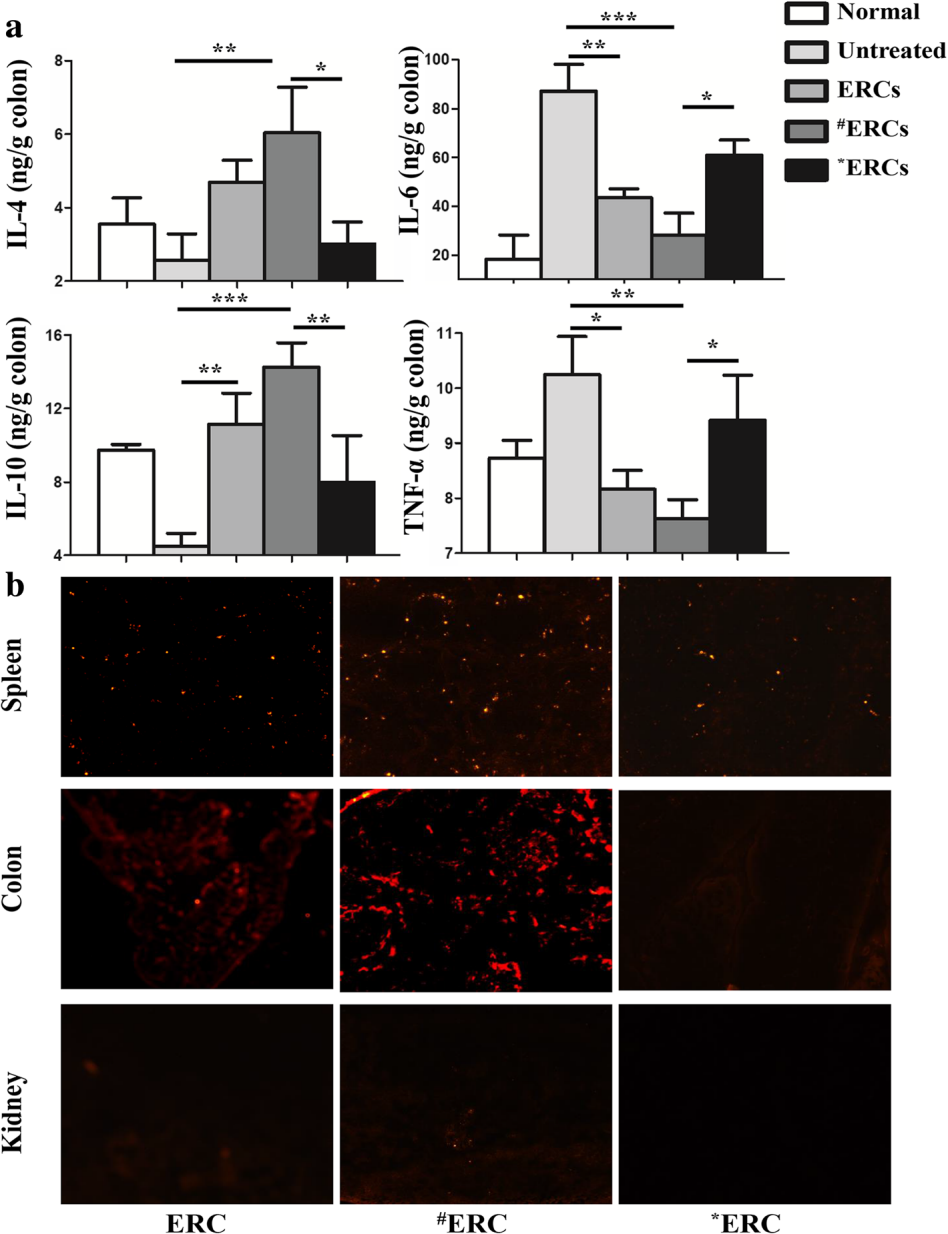
CXCR4 high-expressing ERCs modulated the balance of anti-inflammatory and pro-inflammatory cytokines in the injured colon, and CM-Dil-positive SDF-1-treated ERCs were found to be clustered. **a** Homogenized the colon samples and harvested the supernatants. The concentration of TNF-α, IL-4, IL-6, and IL-10 was measured by ELISA. **b** CM-Dil-positive SDF-1-treated ERCs were significantly detected in the injured colon tissue and spleen compared with CM-Dil-positive ERCs, but not in the kidney. ^**#**^ERCs indicated ERCs pretreated with SDF-1. *ERCs indicated inhibition of the function of SDF-1 by AMD3100. The *p* value was determined by one-way ANOVA. ***p < 0.001, **p < 0.01, *p < 0.05

To further confirm the levels of TNF-α, IL-4, IL-6, and IL-10 in the colon, we measured these cytokines by ELISA. The SDF-1-pretreated ERCs group markedly increased the anti-inflammatory IL-4 and IL-10 cytokine levels, as compared with both the untreated group and AMD3100-pretreated ERCs group (Fig. 6a; SDF-1-pretreated ERCs group vs. untreated group: IL-4 *p* < 0.01, IL-10 *p* < 0.001; SDF-1-pretreated ERCs group vs. AMD3100-pretreated ERCs group: IL-4 *p* < 0.05, IL-10 *p* < 0.01). As compared with the cytokine profiles in both the untreated group and AMD3100-pretreated ERCs group, the pro-inflammatory TNF-α and IL-6 cytokine levels were significantly decreased in the SDF-1-pretreated ERCs group (Fig. 6a; SDF-1-pretreated ERCs group vs. untreated group: IL-6 *p* < 0.001, TNF-α *p* < 0.01; SDF-1-pretreated ERCs group vs. AMD3100-pretreated ERCs group: IL-6 *p* < 0.05, TNF-α *p* < 0.05). These results demonstrated that SDF-1-pretreated ERC-based therapy not only increases the level of anti-inflammatory cytokines, but also inhibits the level of pro-inflammatory cytokines in DSS-induced colitis in mice.

### Tracking of SDF-1-pretreated ERCs in vivo

To determine the in vivo migration of CM-Dil-labeled ERCs, the injured colon, spleen, and native kidney were collected from the colitis mice 24 h after ERC administration. As shown in Fig. 6b, CM-Dil-labeled SDF-1-pretreated ERCs were mainly detected in the injured colon and the spleen, but not in the native kidney by fluorescence microscopy.

## Discussion

The present study demonstrated that SDF-1 effectively increased the CXCR4 expression on ERCs. In vitro, SDF-1-pretreated ERCs with a high level of CXCR4 expression had a much higher ability in reducing the generation of DCs and stimulating the generation of M2 and Tregs than unaltered ERCs. Furthermore, the results from in vivo experiments not only were similar to our previous report, but also demonstrated that CXCR4 high-expressing ERCs more significantly alleviated experimental colitis, which associated with a decrease in pro-inflammation cytokines (IL-6 and TNF-α) and/or with upregulation of anti-inflammation cytokines (IL-4 and IL10) in the colon. These therapeutic effects of ERCs were suppressed by AMD3100 that blocked the function of SDF-1/CXCR4 axis.

UC is mainly mediated by Th2 immune response and causes inflammation of the colon tissue [31]. Our previous study has reported that ERCs could effectively attenuate experimental colitis in mice [20]. ERCs are a new type of mesenchymal-like stromal cells derived from menstrual blood and highly express CD90 and CD105 but lack the expression of CD34 and CD45 on the cell surface [20, 21, 32]. We have previously demonstrated the therapeutic efficacy of ERCs in various animal models such as acute liver injury, renal ischemia-reperfusion injury, colitis, and transplant rejection [17, 19, 33, 34]. By using an in vivo cell tracking, we have found that ERCs are not rejected by the recipients and mainly migrate to injured tissues/organs for tissue repair and to a lymphoid organ such as the spleen and lymph node for immune cell education [18]. Therefore, the homing mechanisms of these MSCs are not fully understood. Both BMSC and ERC supernatants contain high concentrations of SDF-1 [25] that acts through its CXCR4 receptor to form the SDF-1/CXCR4 axis and modulates several diverse stem/stromal cell functions [35]. However, it has been reported that exogenous BMSCs do not express relevant amounts of CXCR4 [36], which would impede efficient migration and homing of BMSCs to the injured colon tissue.

We have hypothesized that enhancing the colonization of ERCs in the damaged tissues will improve the therapeutic effect of these cells. SDF-1 is a unique chemokine that is highly conserved in mammals [37]. This molecule plays an essential role in the survival of the embryo and is indispensable for stem cells homing via binding to and signals through CXCR4 [37]. SDF-1 could stimulate the chemotactic response and lead to the changes in cell adhesion and cell secretion, and then guiding them to migrate to a high SDF-1 concentration gradient across the basement membrane of the endothelium [38]. SDF-1α has been widely confirmed as a homeostatic rather than an inflammatory chemokine that promotes tissue repair in various organs [37, 39, 40]. However, it has been reported that when the stem cells are cultured in vitro, the expression of CXCR4 on the surface of stem cells is decreased, which affects the binding effect with SDF-1, thereby affecting the efficacy of ERCs [41]. There is evidence that the expression levels of CXCR4 on the bone marrow cell surface determine the efficiency of cell homing [42]. Our research for the first time used the in vitro assays to demonstrate that co-culture of different concentrations of SDF-1 with ERCs promoted the expression of CXCR4 on the surface of ERCs.

Macrophages and DCs as the two crucial antigen-presenting cells in the innate immune system play important roles in antigen presentation and phagocytosis. Their differentiation and maturation will further impact the formation and differentiation of adaptive immune cells [43, 44]. In the experimental colitis model, activated DCs produce numerous pro-inflammatory cytokines including TNF-α, IL-1β, IL-6, and IL-12 and express high levels of co-stimulatory molecules including CD80 and CD86 [45]. Activated type 2 macrophage (M2) may act as non-inflammatory scavengers of bacteria [46]. Accordingly, MSCs could promote the increasing of M2 and Tol-DC in injured colons tissue, which will have beneficial effects on the treatment of UC [47]. Our previous study showed that ERCs may suppress maturation of DCs and change their secretion profile towards tolerogenic phenotype, resulting in a decrease in the production of pro-inflammatory TNF-α, IL-1β, and IL-6 and/or an increase in the production of anti-inflammatory IL-10, which together lead to the intensified Treg and Breg cells [20]. The ERCs can also directly change the cytokine profile of CD4^+^ T cells by increasing the production of Th2 cytokines such as IL-4 and IL-10 [17]. It is noteworthy that in this study, we found that SDF-1-pretreated ERCs significantly decreased the generation of DCs, enhanced the generation of M2 and Tregs both in vivo and in vitro, increased the anti-inflammatory cytokines, and/or reduced pro-inflammatory cytokine infiltration in injured colon tissue. All these imply that SDF-1 pretreatment does not attenuate the immunomodulation of ERCs in mice with colitis.

However, when the function of SDF-1/CXCR4 axis was inhibited by AMD3100, the therapeutic effects of ERCs were eliminated. It has been reported that hypoxia-inducible factor-1 (HIF-1) in endothelial cells induces the expression of SDF-1, which increases the ability of the adhesion, migration, and homing of circulating CXCR4^+^ progenitor cells to the damaged tissue [48]. Blocking SDF-1 in damaged tissue or CXCR4 on circulating cells prevents progenitor cells from being recruited to the site of injury [48]. The data from the current study suggested that the SDF-1/CXCR4 system might play a role in the development of UC. The symptoms of the DSS-induced colitis model are indicated by the weight loss, bloody stools, and colon shortening, similar to the UC patients [49]., and we showed in this experimental model that the administration of ERCs pretreated with SDF-1 further reduced both DAI score and inflammatory cell infiltration. Enhanced SDF-1/CXCR4 axis may direct or facilitate the recruitment of ERCs to the injured colon tissue and lymphoid tissue such as the spleen, as shown by cell tracking in vivo. As a result, the therapeutic effect of ERCs is improved.

The therapeutic potential of ERCs for a range of clinical applications has been investigated in several preclinical and clinical trials, in which no immunological reactions or treatment-related side effects are noticed during the follow-up [16, 50, 51]. In this study, we demonstrated the anti-inflammatory and immunosuppressive effects of ERCs in reducing colitis in mice, which may make a significant prospect in future clinical treatment for inflammatory bowel disease.

## Conclusions

In this study, we demonstrate that the chemotactic interaction of SDF-1/CXCR4 system promotes homing of ERCs to the impaired colon tissue in the DSS-induced colitis model in mice. The immunoregulatory or tolerogenic responses induced by the ERCs pretreated with SDF-1 could support damaged organ repair. In addition, SDF-1 effectively enhances the expression of CXCR4 on the surface of ERCs and thereby markedly improves the therapeutic effect of ERCs in alleviating colitis. The potential use of SDF-1-pretreated ERCs in future clinical use remains further investigation.

## Data Availability

The dataset supporting the conclusions of this article is included within the article.

## Abbreviations

AMD3100: CXCR4 antagonist
ANOVA: Analysis of variance
APCs: Antigen-presenting cells
BSA: Bovine serum albumin
CD: Crohn’s disease
CXCR4: C-X-C chemokine receptor type 4
DAI: Disease Activity Index
DMEM: Dulbecco’s modified Eagle’s medium
DSS: Dextran sulphate sodium
Elisa: Enzyme-linked immunosorbent assay
ERCs: Endometrial regenerative cells
FACS: Fluorescence-activated cell sorting
FBS: Fetal bovine serum
H&E: Hematoxylin and eosin
IBD: Inflammatory bowel disease
IL: Interferon
LESCs: Limbal epithelium stem cells
LPS: Lipopolysaccharide
LSD: Least significant difference
M2: Type 2 macrophage
MSCs: Mesenchymal stem cells
OD: Optical density
PBS: Phosphate-buffered saline
SDF-1: Stromal cell-derived factor-1
SEM: Standard error of the mean
Th: T helper
TNBS: 2,4,6-Trinitrobenzene sulfonic acid
TNF: Tumor necrosis factor
Tol-DCs: Tolerogenic dendritic cells
Tregs: Regulatory T cells
UC: Ulcerative colitis
